# Acute kidney injury as an independent risk factor for unplanned 90-day hospital readmissions

**DOI:** 10.1186/s12882-016-0430-4

**Published:** 2017-01-06

**Authors:** Simon Sawhney, Angharad Marks, Nick Fluck, David J. McLernon, Gordon J. Prescott, Corri Black

**Affiliations:** 1 University of Aberdeen, Institute of Applied Health Sciences, Aberdeen, UK; 2 NHS Grampian, Aberdeen, UK; 3 Farr Institute@Scotland, Aberdeen, UK

**Keywords:** Acute kidney injury, Acute renal failure, Patient readmission, Heart failure, Patient discharge, Decision support techniques, Prediction model, Clinical decision-making, Epidemiology, Prognosis

## Abstract

**Background:**

Reducing readmissions is an international priority in healthcare. Acute kidney injury (AKI) is common, serious and also a global concern. This analysis evaluates AKI as a candidate risk factor for unplanned readmissions and determines the reasons for readmissions.

**Methods:**

GLOMMS-II is a large population cohort from one health authority in Scotland, combining hospital episode data and complete serial biochemistry results through data-linkage. 16453 people (2623 with AKI and 13830 without AKI) from GLOMMS-II who survived an index hospital admission in 2003 were used to identify the causes of and predict readmissions. The main outcome was “unplanned readmission or death” within 90 days of discharge. In a secondary analysis, the outcome was limited to readmissions with acute pulmonary oedema. 26 candidate predictors during the index admission included AKI (defined and staged 1–3 using an automated e-alert algorithm), prior AKI episodes, baseline kidney function, index admission circumstances and comorbidities. Prediction models were developed and assessed using multivariable logistic regression (stepwise variable selection), C statistics, bootstrap validation and decision curve analysis.

**Results:**

Three thousand sixty-five (18.6%) patients had the main outcome (2702 readmitted, 363 died without readmission). The outcome was strongly predicted by AKI. Multivariable odds ratios for AKI stage 3; 2 and 1 (vs no AKI) were 2.80 (2.22–3.53); 2.23 (1.85–2.68) and 1.50 (1.33–1.70). Acute pulmonary oedema was the reason for readmission in 26.6% with AKI and eGFR < 60; and 4.0% with no AKI and eGFR ≥ 60. The best stepwise model from all candidate predictors had a C statistic of 0.698 for the main outcome. In a secondary analysis, a model for readmission with acute pulmonary oedema had a C statistic of 0.853. In decision curve analysis, AKI improved clinical utility when added to any model, although the incremental benefit was small when predicting the main outcome.

**Conclusions:**

AKI is a strong, consistent and independent risk factor for unplanned readmissions – particularly readmissions with acute pulmonary oedema. Pre-emptive planning at discharge should be considered to minimise avoidable readmissions in this high risk group.

**Electronic supplementary material:**

The online version of this article (doi:10.1186/s12882-016-0430-4) contains supplementary material, which is available to authorized users.

## Background

Reducing unplanned readmissions after hospital discharge is an international priority for modern healthcare systems [1–3]. Readmission rates are driven by a mixture of health and social factors and some are potentially avoidable [4, 5]. Globally, performance indicators, financial penalties, safety initiatives and prediction tools have been developed to reduce unplanned readmissions [1, 2]. Clinical prediction tools combine available patient characteristics to predict a diagnostic or prognostic outcome [6]. Tools that predict whether patients leaving hospital are at a high risk of unplanned readmission would be helpful for the delivery of safe patient-centred care. However, current tools are limited by the inclusion of risk factors that are not widely accessible in routine clinical practice [7–9] (e.g., critical care scores or subjective social assessments). These limitations preclude a more general use of risk prediction tools for unplanned readmissions in clinical practice.

Acute kidney injury (AKI) is an abrupt change in kidney function, usually measured by a rising serum creatinine. As AKI is common across all hospital settings (1 in 7 hospital admissions), serious [10], and objectively measurable in a standardised fashion (using automated e-alerts) [11], it is a promising novel candidate risk factor for readmission. Good practice in AKI frequently requires rehydration with fluids and temporary discontinuation of cardiac medications until a patient improves [12]. People with AKI transition through multiple care providers and therefore good communication and awareness is needed to ensure that avoidable complications (e.g., overload, cardiovascular complications) do not occur. For this reason, AKI is now also the target of quality initiatives, including efforts to improve handovers at hospital discharge [13].

Previous work suggests an association between hospital AKI and increased hospital readmissions, but with methodological limitations. In one U.S study, the authors were unable to distinguish between planned and unplanned admissions. Pre-hospital creatinine values were also unavailable, which meant that only a minority subset of AKI (those who deteriorated during admission) could be analysed and AKI severity could not be staged [14]. A second U.S. study also associated AKI with readmissions, but was limited to survivors of AKI occurring in intensive care [15]. A third recently reported study from Canada showed a 1.5-fold increase in 30 day readmissions among those with AKI in a propensity-matched cohort, but only a subset comprising those with more severe AKI recorded using ICD-10 codes were represented [16].

In this analysis, we evaluated the clinical utility of hospital AKI of all severities as a candidate risk factor for predicting and reducing unplanned hospital readmissions. We assessed whether AKI was an independent risk factor that could be used to guide decisions either in isolation or as part of a parsimonious clinical risk prediction tool. We also assessed whether the reasons for readmission were different for those with and without AKI, which would motivate the consideration of pre-emptive care plans at hospital discharge after AKI.

## Methods

### Population

This study includes all patients from the Grampian Laboratory Outcomes Morbidity and Mortality Study-II (GLOMMS-II) who were admitted to hospital in 2003 and survived to discharge (*n* = 16453). GLOMMS-II is a population cohort linking national and regional data sources for a single UK health authority (1999–2009). It includes routine hospital administrative data and the complete serial renal biochemistry profile for each patient [17–20]. Crucially for renal disease cohorts, all biochemistry is provided by a single biochemistry service, regardless of clinical location (inpatient, outpatient, community). This minimises any loss of baseline and follow up data and avoids selection biases in patient recruitment [20, 21]. Linkage to hospital episode data and the Scottish Renal Registry (SRR) provided mortality, admission episodes, morbidity events and chronic renal replacement therapy (RRT). Patients receiving chronic RRT prior to index hospital admission were excluded. The study had Regional Ethics Committee approval (14/NW/1371). Data were hosted and managed by Grampian Data Safe Haven [22].

### Outcomes – Unplanned readmission or death within 90 days

The main outcome of interest was unplanned readmission or death within 90 days of discharge. We chose 90 days, because current international AKI guidelines recommend a reassessment at 3 months after AKI for the evaluation of future risk [23]. A distinction between unplanned and planned admissions is possible in Scotland because elective and emergency hospital episodes are specifically distinguished in the Scottish Morbidity Record (SMR01) by trained coders [24]. As 1.1% died within 90 days without first being readmitted, this more severe endpoint was combined with readmission for the logistic regression. As a sensitivity analysis, we also analysed readmission using a multinomial approach (i.e., readmission vs alive and not readmitted; and death without readmission vs alive and not readmitted), which yielded similar results. As additional sensitivity analyses, we also generated models for the main outcome at 30 and 60 days. Finally, because we identified a substantial increase in readmissions due to acute pulmonary oedema (a potentially modifiable reason) among those with AKI, we conducted a secondary analysis of the outcome restricted to readmission with acute pulmonary oedema.

### Follow up

Follow up was from the date of discharge from the original (index) admission until the next emergency readmission or death. The index admission, as previously described, was the first admission with AKI, or last admission without AKI in 2003 [20]. 94.6% of the study population had evidence of follow-up (e.g., linkage to blood tests) up to or beyond the end of the study. Of the remainder, as migration out of Grampian was negligible for the period and age-mix of the cohort [25], those without follow-up beyond the end of the study were assumed to be alive without achieving the main outcome.

### Covariates and candidate predictors

Based on previous studies [7, 9, 14], a combination of renal measures, comorbidities, social measures and admission circumstances were included as candidate predictors. Renal measurements included AKI severity (stages 0–3, 0 being no AKI) for an index episode in 2003, the presence of AKI episodes in the prior 91–1095 days (i.e., AKI in the 3 years prior to the baseline lookback period), baseline estimated glomerular filtration rate (eGFR) and the presence of a >20% worsening of serum creatinine from baseline to hospital discharge (i.e., non-recovery). AKI and baseline kidney function were determined using the “Kidney Disease: Improving Global Outcomes” (KDIGO) criteria [23, 26]. We used a KDIGO-based AKI e-alert definition to identify all discrete AKI episodes lasting up to 90 days from 2000 until the end of 2003 [20]. A summary of this AKI definition is also provided in Table 1 with more detail available elsewhere [20]. AKI severity was the highest stage achieved (1–3) within each AKI episode period with respect to the baseline identified at the point of identification of each new AKI episode. The rolling lookback period ensured that the baseline creatinine was updated between AKI episodes so that further rises in creatinine after an AKI episode could be distinguished either as recurrent AKI (further rapid rises above a prior AKI episode and meeting KDIGO cirteria) or CKD progression/non-recovery (elevated creatinine following a prior AKI episode but no actual acute rise meeting KDIGO cirteria). Baseline eGFR was reported using the Chronic Kidney Disease Epidemiology Collaboration (CKD-EPI) creatinine equation [27].

**Table 1:** KDIGO-based acute kidney injury criteria for this study (as described in [20])

AKI criteria	Definition
Index AKI episode (lasting up to 90 days in duration)	Serum creatinine ≥1.5 times higher than the median of all creatinine values 8-90 days ago; or 91–365 days ago if no tests between 8 and 90 days; or serum creatinine ≥1.5 times higher than the lowest creatinine within 7 days; or serum creatinine >26 μmol/L higher than the lowest creatinine within 48 h
Prior AKI episode	Any episode occurring 91-1095 days prior to index episode

Comorbidities were the “international classification of diseases” (ICD-10) codes for Charlson comorbidities from the 5 years prior to admission as previously described and validated [28]. Social and demographic measures included age, sex, residential care (long-term care home or skilled nursing home), deprivation and rural home location as previously described [20, 29]. Metrics of admission circumstances were the number of hospital admissions in the past year, length of hospital stay for the index admission, emergency or elective admission, and admission to a medical ward or intensive care. For the causes of readmission, ICD-10 diagnoses for the readmission episode were also recorded. Based on previous work and validation studies these were acute coronary syndrome (with or without infarction) (I21-I22, I20) [30, 31]; cerebrovascular disease (G45, I60-67) [32–34]; lower respiratory tract infection (pneumonia or bronchitis) (J10-18, J20-22) [35, 36]; and acute pulmonary oedema in the context of heart failure (I50) [37, 38].

### Statistical analyses

We plotted Kaplan-Meier curves for readmission-free survival with risk tables showing numbers alive, readmitted and dead up to 1 year after hospital discharge.

To compare the reasons for hospital readmission in those with and without AKI, we recorded the readmission ICD-10 codes for readmission diagnoses. Based on prior research [14], and the recognised role of fluids and cardiac medications in AKI [12], we reported four specific diagnoses: acute coronary syndrome, cerebrovascular event, lower respiratory tract infection, acute pulmonary oedema.

We performed univariable and multivariable logistic regression to assess the association of each candidate predictor with 90 day readmission or death. We performed multivariable logistic regression using a full model containing all candidate predictors. To determine a “best stepwise model”, we then used all candidate predictors with stepwise backwards elimination of predictors with a *p*-value ≥0.01 [6, 39, 40]. This *p*-value threshold was chosen to approximate to the Bayesian Information Criterion (BIC) for the large sample size of the analysis [41, 42]. We also developed models using the same stepwise procedure but limiting candidate predictors to administrative data only (including age); age and renal biochemistry only; age alone; and AKI alone. Based on prior knowledge and graphical inspection, we modelled age and eGFR continuously using linear and quadratic terms [43]. We repeated this modelling procedure for an additional outcome limited to readmissions with acute pulmonary oedema, and for outcomes at 30 and 60 days.

### Assessment of model performance

We assessed model performance by testing discrimination and calibration. Discrimination measures how well a model distinguishes between those with and without an outcome. We calculated the area under the receiver operating characteristic curve (AUC), which can be considered equivalent to a C-statistic [44]. The value of a C statistic lies between 0.5 and 1; 0.5 meaning that the model is no better than a coin toss at discrimination and 1 meaning perfect discrimination. Pairwise comparison of C statistics for different models was performed as previously outlined elsewhere [45, 46]. Calibration is a measure of how well the predicted probabilities of an outcome from a model agree with the observed probabilities of the outcomes. We developed a calibration plot of mean observed probability vs mean predicted probability of outcomes within tenths of increasing predicted risk. All points lying on a calibration slope of 1 indicates perfect agreement, and a slope of less than 1 indicates over-fitting of the model [6]. We also performed a Hosmer-Lemeshow goodness-of-fit test to assess for a statistically significant difference between observed and predicted values, standardised for sample size using a method described elsewhere [47].

Validation of a model using the same data as used to develop the model (known as apparent validation) usually results in optimistic measures of performance. Therefore, we performed internal validation of the best stepwise model using bootstrap resampling. We generated 500 bootstrapped datasets with replacement from the original dataset. For each bootstrapped sample we applied the same backward selection modelling process used to derive the original best stepwise model. The C statistic was calculated for each of the 500 bootstrapped models both in the bootstrap data set and the original data set. The difference between the two C statistics for each sample was found and averaged over the 500 samples. This average difference indicated the optimism of the C statistic in the original model and is an estimate of internal validity. This procedure enabled us to provide a C statistic and calibration slope corrected for model optimism [6]. As stepwise procedures can lead to instability in variable selection we also recorded the variable inclusion frequencies for each of the bootstrap models using the backwards elimination procedure with a p ≥ 0.01 threshold [42, 48].

### Model application

Even if a risk factor improves model discrimination and calibration, a model may not result in better decisions. Decision curve analysis is a recent method of assessing the clinical usefulness of different models at an appropriate threshold for clinical use [49]. We used decision curve analysis to compare each model and also strategies of “predict all” or “predict none”.

Decision curve analysis is a plot of the “*net benefit*” against “*threshold probabilities”*. Such a plot identifies the range of threshold probabilities for which the model is of clinical value i.e., can be used to guide decisions. The *threshold probability* indicates the cut-off for classifying a prediction as positive or negative for an outcome [50]. An acceptable threshold may differ for clinicians and patients making decisions in different clinical contexts. A threshold of close to zero would imply that false-positive predictions are acceptable to ensure that no patients are missed. A higher threshold would involve targeting only higher risk patients with fewer false-positives. *Net benefit* measures the trade-off between true-positives and false-positives in a prediction model at different threshold probabilities. It is a sum of true-positive minus false-positive predictions weighted by the threshold probability as described in the equation below [49].

$$ Net\  benefit=\left(\frac{true\  positive}{total\  sample\  size}\right) - \left[\left(\frac{false\  positive}{total\  sample\  size}\right) \times \left(\frac{threshold\  probability}{1- threshold\  probability}\right)\right] $$

The model with the highest net benefit at a given threshold, has the greatest clinical value. At a threshold probability of zero a policy of targeting all patients would be of greatest value as there would be no penalty from false-positives. At higher thresholds, an alternative approach guided by a prediction model may provide greater benefit. In this study, if a clinician wished to identify patients with higher than average risk of readmission, this would correspond to a threshold probability of 0.2–0.4 and a prediction model would need to show greater net benefit over this pre-specified range.

Decision curve analysis comprehensively compares models across all thresholds, however we also calculated the integrated discrimination improvement (IDI) and categorical net reclassification improvement (NRI) with categories of low, medium and high risk using a thresholds of 0.1 and 0.3 for the main outcome and 0.01 and 0.1 for the outcome limited to readmissions with pulmonary oedema for those familiar with these alternative metrics [51, 52].

Statistical analysis was performed in Stata SE version 13 using “*dca*”, “*incrisk*” and “*roccomp*” packages for the assessments of model performance [45, 53–55]. We also developed a web-based application to illustrate how risk predictions changed in the presence of AKI. This was performed in R using the package “*shiny*” and “*personograph*”[56–58].

## Results

### Cohort characteristics

**Fig. 1:**
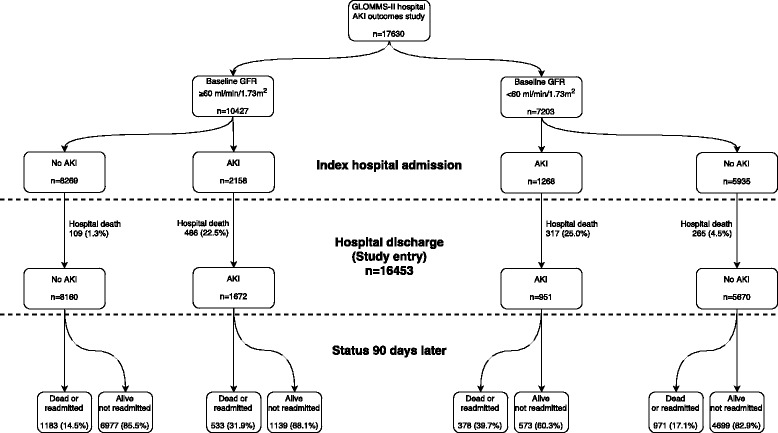
Description of the cohort developed for this study from those surviving to hospital discharge, and the overall 90 day outcomes broken down by AKI status and baseline eGFR

Cohort formation and characteristics of 16453 patients surviving an index hospital admission (2623 with AKI and 13830 without AKI), with and without readmission or death by 90 days, are described in Fig. 1 and Table 2. Univariable odds ratios are provided in the final column of Table 2. Three thousand sixty five of 16453 patients (18.6%) were readmitted or died within 90 days of hospital discharge (including 363 deaths occurring without readmission and 269 readmissions with pulmonary oedema). Those with readmission or death were older, more frequently in residential care, had more previous hospital admissions, longer hospital stay at index admission and more comorbidities. They also experienced more AKI during index admission, more prior AKI episodes, and had worse kidney function at baseline and at discharge.

**Table 2:** Cohort characteristics

	90 day readmission or death (%)		90 day readmission free survival (%) baseline impairment		Univariable odds ratio (95% CI)	
N	3065		13388			
Age (median, IQR)	74	(62–83)	69	(55–79)	1.20 (/10 years)	(1.17–1.23)
Male sex	1372	(44.8)	5770	(43.1) ())	1.07	(0.99–1.16)
Residential care	277	(9.0)	471	(3.5)	2.72	(2.34–3.18)
Deprived (highest quintile)	298	(9.7)	1088	(8.1)	1.22	(1.06–1.39)
Rural (settlement <3000)	735	(24.0)	3720	(27.8)	0.82	(0.75–0.90)
Admission context						
No admissions past year^a^	1896	(61.9)	10461	(78.1)	1.38 (/admission)	(1.34–1.43)
1 admission past year	593	(19.3)	1872	(14.0)		
2 admissions past year	244	(8.0)	620	(4.6)		
3+ admissions past year	332	(10.8)	435	(3.2)		
Length of stay (median, IQR)	7	(2–16)	3	(1–9)	1.05 (/7 days)	(1.04–1.06)
Emergency admission	2326	(75.9)	7760	(58.0)	2.28	(2.09–2.50)
Medical ward admission	1889	(61.6)	6570	(49.1)	1.67	(1.54–1.81)
Intensive care admission	98	(3.2)	366	(2.7)	1.18	(0.94–1.47)
Renal function						
No AKI	2154	(70.3)	11676	(87.2)	(reference group)	
AKI stage 1	528	(17.2)	1190	(8.9)	2.41	(2.15–2.69)
AKI stage 2	233	(7.6)	341	(2.5)	3.70	(3.12–4.40)
AKI stage 3	150	(4.9)	181	(1.4)	4.49	(3.60–5.60)
No prior AKI episodes^a^	2526	(82.4)	12188	(91.0)	1.71 (/episode)	(1.58–1.85)
1 prior AKI episode	414	(13.5)	1000	(7.5)		
2+ prior AKI episodes	125	(4.1)	200	(1.5)		
Baseline eGFR (median, IQR)^a^	63	(48–83)	66	(52–87)	0.94 (/10 ml/min/1.73 m^2^)	(0.93–0.96)
Baseline eGFR 0–29	213	(6.9)	502	(3.7)		
Baseline eGFR 30–44	423	(13.8)	1461	(10.9)		
Baseline eGFR 45–59	713	(23.3)	3309	(24.7)		
Baseline eGFR ≥60	1716	(56.0)	8116	(60.6)		
Discharge creatinine 20% > baseline	520	(17.0)	1167	(8.7)	2.14	(1.91–2.39)
Comorbidity						
Cancer	410	(13.4)	973	(7.3)	1.97	(1.74–2.23)
Cardiac failure	317	(10.3)	592	(4.4)	2.49	(2.16–2.88)
Cerebrovascular disease	231	(7.5)	580	(4.3)	1.80	(1.54–2.11)
Dementia	96	(3.1)	163	(1.2)	2.62	(2.03–3.39)
Diabetes	336	(11.0)	776	(5.8)	2.00	(1.75–2.29)
Hemiplegia	28	(0.9)	71	(0.5)	1.73	(1.11–2.68)
Liver disease	59	(1.9)	156	(1.2)	1.66	(1.23–2.25)
Myocardial infarction	257	(8.4)	638	(4.8)	1.83	(1.57–2.13)
Peptic ulcer disease	81	(2.6)	278	(2.1)	1.28	(1.00–1.64)
Peripheral vascular disease	162	(5.3)	452	(3.4)	1.60	(1.33–1.92)
Pulmonary disease	346	(11.3)	704	(5.3)	2.29	(2.00–2.62)
Rheumatic disease	82	(2.7)	289	(2.2)	1.25	(0.97–1.60)

*Abbreviations: AKI* acute kidney injury, *CI* confidence interval, *eGFR* estimated glomerular filtration rate, *IQR* inter-quartile range

^a^Modelled here linearly per 10 ml/min/1.73 m^2^ increase and reported in categories for clarity

### Reasons for readmission after AKI

**Fig. 2:**
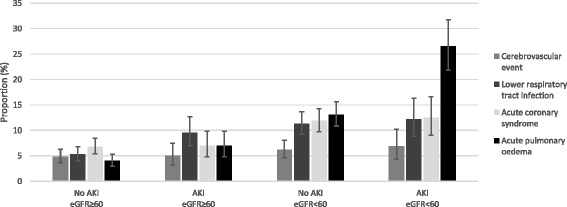
Reasons for unplanned hospital readmission among those people in the cohort readmitted within 90 days of hospital discharge

Reasons for readmission grouped by AKI and baseline eGFR are summarised in Fig. 2. There was little difference in readmissions due to cerebrovascular episodes, and a modest increase in readmissions due to lower respiratory tract infection or acute coronary episodes among those with eGFR < 60 ml/min/1.73 m^2^. However, there was a substantial increase in readmissions due to acute pulmonary oedema in those with AKI and eGFR < 60 ml/min/1.73 m^2^ (respectively 26.6%; 13.1%; 7.0 and 4.0% for AKI and eGFR < 60; no AKI and eGFR < 60; AKI and eGFR ≥ 60; no AKI and eGFR ≥ 60 ml/min/1.73 m^2^). This trend was the same whether acute pulmonary oedema was used in any diagnostic position (as above) or restricted to the main diagnosis.

### Relationship between AKI and unplanned readmission or death

**Fig. 3:**
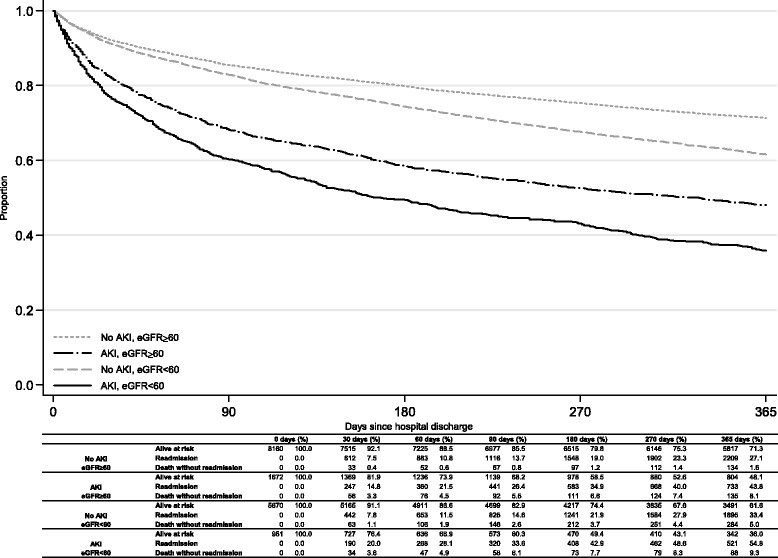
Unadjusted curves of readmission-free survival with risk table of death and readmission by AKI status and baseline eGFR

Figure 1 describes the status of patients over the first 90 days after discharge from index admission. There were 16453 patients who survived an index hospital admission. Patients with AKI (vs no AKI) and baseline eGFR < 60 (vs eGFR ≥ 60 ml/min/1.73 m^2^) had poorer outcomes, with greater occurrence of unplanned readmission or death. Figure 3 is a Kaplan-Meier plot of readmission-free survival stratified by AKI and baseline eGFR. At all time-points up to 1 year, patients with AKI had poorer outcomes than those without AKI and patients with baseline eGFR < 60 had poorer outcomes than those with baseline eGFR ≥ 60 ml/min/1.73 m^2^.

### AKI as an independent predictor of unplanned readmission or death

Table 3 summarises multivariable logistic regression using all candidate predictors (full model), and using a stepwise regression (best stepwise model). AKI (staged 0–3) independently predicted readmission. Four comorbidities (cancer, cardiac failure, diabetes and chronic pulmonary disease) were also present in the best model. AKI was one of the most consistently selected variables, present in 100% of 500 bootstrapped models. Age, residential care, number of previous admissions, emergency admission and cancer were also selected in 100% of bootstrapped models. In sensitivity analysis, this did not change if those who died without readmission were not included in the outcome. Models for 60 and 30 day outcomes were also similar (Additional file 1: Table S1). When the outcome for analysis was restricted to readmissions with pulmonary oedema, age, AKI and history of previous cardiac failure were the most consistently selected variables (Additional file 1: Table S2).

**Table 3:** Stepwise model of unplanned 90 day readmission or death after hospital discharge

	Full model			Best stepwise model			
	OR	95% CI	*p*-value	OR	95% CI	*p*-value	Variable Inclusion %^b^
Characteristics							
Age (per 10 years)	1.19	(1.14–1.24)	<0.001	1.17	(1.13–1.21)	<0.001	100
Age term squared	1.00	(0.99–1.02)	0.388				12.4
Male sex	1.08	(1.00–1.18)	0.061				26.4
Residential care	1.63	(1.36–1.94)	<0.001	1.37	(1.42–1.98)	<0.001	100
Deprived (highest vs all other quintiles)	1.11	(0.96–1.29)	0.150				15.4
Rural (settlement <3000)	0.88	(0.80–0.97)	0.008	0.86	(0.78–0.94)	0.001	65.2
Admission context							
Admissions in prior 1 year (per admission)	1.21	(1.17–1.25)	<0.001	1.23	(1.18–1.27)	<0.001	100
Length of stay (per week)	0.99	(0.98–1.00)	0.242				7.0
Emergency admission	1.81	(1.64–2.01)	<0.001	1.89	(1.72–2.08)	<0.001	100
Medical ward admission	1.09	(0.99–1.19)	0.066				24.6
Intensive care admission	0.88	(0.68–1.13)	0.308				5.2
Renal function							
No AKI	(reference group)						
AKI stage 1	1.57	(1.36–1.80)	<0.001	1.50	(1.33–1.70)	<0.001	100
AKI stage 2	2.35	(1.92–2.88)	<0.001	2.23	(1.85–2.68)	<0.001	100
AKI stage 3	2.95	(2.29–3.80)	<0.001	2.80	(2.22–3.53)	<0.001	100
Prior AKI count (per episode)	1.11	(1.02–1.22)	0.020				47.4
Baseline eGFR linear term^a^	0.89	(0.82–0.96)	0.005	0.87	(0.80–0.94)	<0.001	
Baseline eGFR squared term^a^	1.01	(1.00–1.02)	0.001	1.01	(1.01–1.02)	<0.001	91.0
Discharge creatinine 20% > baseline	0.93	(0.80–1.08)	0.317				6.0
Comorbidity							
Cancer	1.59	(1.38–1.82)	<0.001	1.59	(1.37–1.82)	<0.001	100
Cardiac failure	1.32	(1.12–1.55)	0.001	1.42	(1.21–1.66)	<0.001	88.8
Cerebrovascular disease	1.07	(0.89–1.27)	0.471				6.0
Dementia	1.21	(0.92–1.60)	0.174				13.2
Diabetes	1.33	(1.15–1.54)	<0.001	1.38	(1.19–1.60)	<0.001	92.0
Hemiplegia	0.91	(0.56–1.47)	0.691				0.4
Liver disease	1.19	(0.86–1.66)	0.299				11.2
Myocardial infarction	1.13	(0.95–1.33)	0.170				13.6
Peptic ulcer disease	0.90	(0.69–1.18)	0.464				2.0
Peripheral vascular disease	1.00	(0.82–1.22)	0.994				0.6
Pulmonary	1.44	(1.24–1.67)	<0.001	1.47	(1.27–1.70)	<0.001	99.2
Rheumatic disease	0.92	(0.70–1.20)	0.525				2.4

*Abbreviations: AKI* acute kidney injury, *CI* confidence interval, *eGFR* estimated glomerular filtration rate, *OR* odds ratio

^a^Modelled per 10 ml/min/1.73 m^2^ increase with a combination of linear and quadratic terms. Variable inclusion % applies to the baseline eGFR variable overall

^b^In 500 bootstrapped datasets

### Performance of prediction models

Table 4 reports the predictors that were significant in stepwise regression when the procedure was limited to groups of candidate predictors. Based on model discrimination (C statistic), performance of the best stepwise model (0.698) was no different to a full model containing all predictors (0.699), and showed statistically significant incremental improvement over models limited to administrative data only; renal biochemistry and age; age alone; and AKI alone. The C statistic for the best stepwise model (0.698) was 0.695 after bootstrap correction for optimism. The bootstrap calibration slope was 0.97 (0.89–1.06), showing excellent agreement at all but the very highest levels of predicted risk on a calibration plot (Additional file 2: Figure S1). For the model limited to readmissions with acute pulmonary oedema, the C statistic was substantially larger (0.853; 0.845 after bootstrap correction for optimism), with more substantial incremental improvements over other models (Additional file 1: Table S3). Again, the bootstrap calibration slope was not significantly different from 1 (0.90, 0.70–1.14). Further details of the calibration of both models is provided in Additional file 2: Figure S1, Additional file 3: Figure S2, Additional file 4: Figure S3 and Additional file 5: Figure S4.

**Table 4:** Comparison of prediction models and model discrimination

	Full model	Best stepwise model	Administrative data only model	Biochemistry + age model	Age alone model	AKI alone
Characteristics						
Age	*	*	*	*	*	
Age term quadratic term	*			*	*	
Male sex	*					
Residential care	*	*	*			
Deprived (highest quintile)	*					
Rural (settlement <3000)	*	*	*			
Admission context						
Admissions in prior 1 year (per admission)	*	*	*			
Length of stay (per week)	*					
Emergency admission	*	*	*			
Medical ward admission	*		*			
Intensive care admission	*					
Renal function						
AKI stages 0–3	*	*		*		*
Prior AKI count (per episode)	*			*		
Baseline eGFR (linear and quadratic)	*	*		*		
Discharge creatinine 20% > baseline	*					
Comorbidity						
Cancer	*	*	*			
Cardiac failure	*	*	*			
Cerebrovascular disease	*					
Dementia	*					
Diabetes	*	*	*			
Hemiplegia	*					
Liver disease	*					
Myocardial infarction	*					
Peptic ulcer disease	*					
Peripheral vascular disease	*					
Pulmonary	*	*	*			
Rheumatic disease	*					
Model C statistic	0.699	0.698	0.685	0.655	0.594	0.587
95% confidence interval	(0.688–0.709)	(0.688–0.709)	(0.675–0.696)	(0.644–0.666)	(0.582–0.605)	(0.578–0.596)
*P*-value for AUC comparison with the next most complex model	-	0.536	<0.001	<0.001	<0.001	0.344

*Abbreviations: AKI* acute kidney injury, *eGFR* estimated glomerular filtration rate

### Model application

**Fig. 4:**
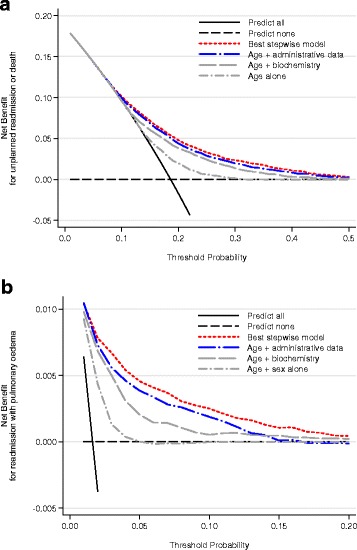
Decision curve analysis comparing the net benefit of prediction models for readmission or death 90 days after discharge (**a**) or for readmission with acute pulmonary oedema 90 days after discharge (**b**)

Figure 4 shows decision curve analysis plots contrasting the net benefit of the different prediction models for the main outcome of death or readmission within 90 days (4A) and for the secondary analysis of readmission with acute pulmonary oedema (4B). At the a priori specified threshold of 0.2–0.4 for the main analysis, all models performed better than predicting readmission in all or no patients. Age provided modest net benefit, with small incremental improvement from adding biochemistry or administrative data to the model and best performance from the best stepwise model from all candidate predictors. For the acute pulmonary oedema model the best stepwise model was again superior to all other models, with larger incremental improvement across all thresholds.

For the main analysis best stepwise model (vs administrative data) the categorical NRI_[0.1, 0.3]_ was +4.6% (+2.7 to +7.0) (event NRI_[0.1, 0.3]_ +2.0% [+0.6 to +3.7]; non-event NRI_[0.1, 0.3]_ +2.7% [+0.6 to +4.2]) and IDI was +0.012 (+0.009 to +0.017) with positive values indicating overall improvement in the prediction probabilities. For the acute pulmonary oedema best stepwise model (vs administrative data) the categorical NRI_[0.01, 0.1]_ was +11.8% (+0.1 to +19.6) (event NRI_[0.01, 0.1]_ +1.5% [−1.9 to +5.4]; non-event NRI_[0.01, 0.1]_ +10.3% [+0.0 to +16.3]) and IDI was +0.009 (+0.001 to +0.024).

Additional file 6: Figure S5 illustrates how predictions change in the presence of AKI, and the accessibility of the data required to generate predictions. This calculator is available on request by contacting the authors. Using the best stepwise model, a 70 year old man with diabetes who is admitted urgently with severe AKI requiring dialysis would have a predicted 90 day risk of readmission or death of 42%. The same man without AKI would have a predicted risk of 21%. In contrast, using the administrative data model (which includes no measure of AKI) the predicted risk would be the same for those with and without AKI (26%).

## Discussion

AKI is common, objectively assessable using serial serum biochemistry (or e-alerts), and associated with increased mortality. Our analysis shows that AKI is also a strong independent predictor of 90 day unplanned readmission or death. Furthermore, there were striking differences in the cause of readmissions between those with and without AKI. Up to 1 in 4 readmissions after AKI were related to acute pulmonary oedema – a potentially modifiable reason for readmission.

Despite being a strong predictor, the incremental improvement in overall predictions after adding AKI to the best alternative model was small, both based on decision curve analysis and on categorical NRI. This is often the case in saturated prediction models [59], but may suggest limited added value of combining renal biochemistry and hospital episode data for general population risk predictions. Nevertheless, our study shows that AKI is still an attractive risk factor in clinical practice because it is common, strongly associated with readmission, and associated with a complication (pulmonary oedema) that could be targeted as part of a pre-emptive discharge plan. This is reinforced by our secondary analysis, which showed that when the outcome was limited to readmissions with acute pulmonary oedema, the absolute performance of the model (C statistic 0.853) and the incremental improvement with the addition of AKI were both greater.

Our analysis is consistent with and extends previous research. Previous work in the U.S. and Canada has associated AKI with readmission, but only studied either a subset of AKI that could be identified using only *inpatient* biochemistry data without distinguishing between planned and unplanned readmissions [14], or the subset of AKI treated in intensive care [15], or the subset of AKI recognised in ICD-10 coding [16]. We extend previous work using a U.K. cohort by capturing all hospital AKI using all biochemistry (inpatient and outpatient), by focusing on unplanned admissions, by using AKI severity and prior AKI episodes as novel predictors and by assessing the incremental benefit of AKI in risk prediction. The bootstrapped C statistic presented in our study (0.695) is consistent with previous UK-based studies predicting 12 month (apparent C statistic 0.685) [8] and 30 day (bootstrapped C statistic 0.699) [9] readmissions. The strongest predictors (age, admission circumstances, cancer, cardiac failure, diabetes and pulmonary disease) also agree with those consistently reported in the literature [7].

Strengths of this analysis include the use of a large unselected population with complete biochemistry and administrative data capture, minimising misclassification due to missing baseline renal data. The linkage of serial renal biochemistry to administrative data demonstrates AKI as a novel and objective predictor that could be reproduced in future research and updated with each admission in real-time clinical practice. The role of AKI and baseline eGFR in readmissions with acute pulmonary oedema was particularly striking. We note previous work has identified heart failure as a common reason for 30 day hospital readmissions [16], and shown that among patients with heart failure, those who develop AKI have more readmissions [60]. This analysis provides the complementary finding that among all hospitalised patients, those with AKI have substantially more emergency readmissions due to acute pulmonary oedema – a potentially preventable reason for readmission [5]. Collectively, these results provide a motivation for improving handovers and medication plans when discharging patients with AKI [13], and for an AKI follow-up clinic [61]. A particular population to focus on could be those with a history of CKD or heart failure, and interventions to evaluate in this group could include volume reassessments, diuretic algorithms and medication reconciliation.

In this study we demonstrated that the use of serial renal laboratory measurements can lead to incremental improvements in clinical risk prediction models. While not the focus of this study, we recognise that a number of other repeatedly measured laboratory parameters (such as discharge sodium, albumin and C-reactive protein) may lead to further incremental improvements. These were not available in this analysis, nor used in previous UK-based readmission prediction tools, and would be an appropriate next step for future research [8, 9]. We also recognise that the role of AKI in our health care system in Scotland may not be generalisable to other regions and health care systems. This may also be the case for other predictors that may differ for health care systems in other countries (e.g., residential care and intensive care). Additional validation would be valuable, because any variation in other regions would also improve our understanding of the circumstances that lead to poorer outcomes after AKI. ICD-10 coding is also subject to variation and misclassification. While sensitive for the clinical diagnosis of heart failure, previous work has shown that the specificity of ICD-10 coding of heart failure can vary depending on whether only the main diagnosis or all diagnoses are included [37, 38]. Nevertheless, we found the trend was the same in both situations. Similarly, because our cohort was originally constructed to observe long-term outcomes in those with kidney disease, the cohort inception was in 2003. We analysed from the cohort inception date because analysis of any later period would have introduced a survivorship bias. While our objective biochemical AKI criteria will not be affected by the cohort dates, recent initiatives to improve the recognition and care of AKI may alter the rates of early readmission. This would be important to reassess, but would be unlikely to materially change the message of our study. Finally, AKI is a clinical diagnosis incorporating the clinical context changes in serum creatinine and urine output. Our identification of AKI involved an algorithm for changes in creatinine in routine data. A problem common to all AKI studies involving large populations is the potential for misclassification bias when blood testing is sometimes infrequent and context is not available to ensure AKI and CKD are classified appropriately.

## Conclusions

Overall, this study indicates that AKI is a strong predictor of unplanned readmissions. Acute pulmonary oedema is a potential driver of the increased readmissions in AKI patients. This raises the possibility that some readmissions after AKI may be avoidable by careful pre-emptive planning after AKI to prevent the development of pulmonary oedema.

## Additional files

Additional file 1: Table S1. Comparison of prediction models and model discrimination for different time points. **Table S2** Stepwise model of unplanned 90 day readmission with pulmonary oedema after hospital discharge. **Table S3** Comparison of prediction models and model discrimination for death or readmission with pulmonary oedema. (DOCX 33 kb)
Additional file 2: Figure S1. Calibration plot for best stepwise prediction model of 90 day readmission or death. Circles represent observed and predicted readmission in deciles of predicted risk. Histograms represent the distributions of patients with (top) and without (bottom) readmission. (PDF 31 kb)
Additional file 3: Figure S2. Calibration plot for best stepwise prediction model of 90 day readmission with acute pulmonary oedema. Circles represent observed and predicted readmission in deciles of predicted risk. Histograms represent the distributions of patients with (top) and without (bottom) readmission. (PDF 40 kb)
Additional file 4: Figure S3. Hosmer-Lemeshow test standardised for sample size for model of 90 day readmission or death with plots of quintiles of observed and predicted risk. (PDF 43 kb)
Additional file 5: Figure S4. Hosmer-Lemeshow test standardised for sample size for model of 90 day readmission with acute pulmonary oedema with plots of quintiles of observed and predicted risk. (PDF 43 kb)
Additional file 6: Figure S5. Illustration of clinical risk prediction model using examples with and without AKI. (PDF 647 kb)

## Abbreviations

AKI: Acute kidney injury
AUC: Area under the receiver operating characteristic curve
BIC: Bayesian Information Criterion
CI: Confidence interval
CKD: Chronic kidney disease
CKD-EPI: Chronic Kidney Disease Epidemiology Collaboration
eGFR: estimated glomerular filtration rate
GLOMMS: Grampian Laboratory Outcomes Morbidity and Mortality Study
ICD: International classification of diseases
IDI: Integrated discrimination improvement
IQR: Inter-quartile range
KDIGO: Kidney disease: improving global outcomes
NRI: Net reclassification improvement
OR: Odds ratio
RRT: Renal replacement therapy
SMR: Scottish morbidity record
SRR: Scottish renal registry
U.S.: United States
