# Supplementary material for: Acute kidney injury as an independent risk factor for unplanned 90-day hospital readmissions
Source: BMC Nephrol. 2017 Jan 6;18:9. doi: 10.1186/s12882-016-0430-4 (PMC5217258; doi:10.1186/s12882-016-0430-4)
Supplement: Additional file 1: Table S1. — Comparison of prediction models and model discrimination for different time points. Table S2 Stepwise model of unplanned 90 day readmission with pulmonary oedema after hospital discharge. Table S3 Comparison of prediction models and model discrimination for death or readmission with pulmonary oedema. (DOCX 33 kb) [file 12882_2016_430_MOESM1_ESM.docx]

Additional table S1 – Comparison of prediction models and model discrimination for different time points for the outcome of 90 day readmission or death after hospital discharge

|  | **90 day outcome** | | **60 day outcome** | | **30 day outcome** | |
| --- | --- | --- | --- | --- | --- | --- |
|  | **Full model** | **Best stepwise model** | **Full model** | **Best stepwise model** | **Full model** | **Best stepwise model** |
| **CHARACTERISTICS** |  |  |  |  |  |  |
| Age | * | * | * | * | * | * |
| Age term quadratic term | * |  | * |  | * |  |
| Male sex | * |  | * | * | * | * |
| Residential care | * | * | * | * | * | * |
| Deprived (highest quintile) | * |  | * |  | * |  |
| Rural (settlement <3000) | * | * | * | * | * |  |
|  |  |  |  |  |  |  |
| **ADMISSION CONTEXT** |  |  |  |  |  |  |
| Admissions in prior 1yr (per admission) | * | * | * | * | * | * |
| Length of stay (per week) | * |  | * |  | * |  |
| Emergency admission | * | * | * | * | * | * |
| Medical ward admission | * |  | * |  | * |  |
| Intensive care admission | * |  | * |  | * |  |
|  |  |  |  |  |  |  |
| **RENAL FUNCTION** |  |  |  |  |  |  |
| AKI stages 0-3 | * | * | * | * | * | * |
| Prior AKI count (per episode) | * |  | * |  | * | * |
| Baseline eGFR (linear and quadratic) | * | * | * | * | * | * |
| Discharge creatinine 20% > baseline | * |  | * |  | * |  |
|  |  |  |  |  |  |  |
| **COMORBIDITY** |  |  |  |  |  |  |
| Cancer | * | * | * | * | * | * |
| Cardiac failure | * | * | * | * | * |  |
| Cerebrovascular disease | * |  | * |  | * |  |
| Dementia | * |  | * |  | * |  |
| Diabetes | * | * | * | * | * | * |
| Hemiplegia | * |  | * |  | * |  |
| Liver disease | * |  | * |  | * |  |
| Myocardial infarction | * |  | * |  | * |  |
| Peptic ulcer disease | * |  | * |  | * |  |
| Peripheral vascular disease | * |  | * |  | * |  |
| Pulmonary | * | * | * | * | * | * |
| Rheumatic disease | * |  | * |  | * |  |
| **Model C statistic** | 0.699 | 0.698 | 0.699 | 0.699 | 0.691 | 0.689 |
| **95% confidence interval** | (0.688-0.709) | (0.687-0.709) | (0.688-0.711) | (0.688-0.711) | (0.678-0.705) | (0.676-0.703) |
| **P-value for AUC comparison with the next most complex model** | - | 0.536 | - | 0.678 | - | 0.025^a^ |
| Abbreviations: AKI, acute kidney injury; eGFR, estimated glomerular filtration rate. | | | | | | |
| ^a^Significant in pairwise comparison but note that cardiac failure was borderline for selection in stepwise procedure (p-value 0.010). If cardiac failure was retained in the selection procedure, the model C statistic would be 0.690 (0.677-0.704), p-value 0.068. | | | | | | |

Additional table S2 – Stepwise model of unplanned 90 day readmission with pulmonary oedema after hospital discharge

|  | **Full model** | | | **Best stepwise model** | | | |
| --- | --- | --- | --- | --- | --- | --- | --- |
|  | **OR** | **95% CI** | **p-value** | **OR** | **95% CI** | **p-value** | **Variable Inclusion %^b^** |
| **CHARACTERISTICS** |  |  |  |  |  |  |  |
| Age (per 10 years) | 1.50 | (1.29-1.75) | <0.001 | 1.41 | (1.23-1.60) | <0.001 | 100 |
| Age term squared | 0.98 | (0.91-1.05) | 0.495 |  |  |  | 3.8 |
| Male sex | 1.52 | (1.17-1.98) | 0.002 | 1.52 | (1.18-1.96) | <0.001 | 73.4 |
| Residential care | 0.53 | (0.30-0.94) | 0.030 | 0.47 | (0.27-0.81) | 0.007 | 54.4 |
| Deprived (highest vs all other quintiles) | 1.49 | (1.00-2.24) | 0.053 |  |  |  | 37.6 |
| Rural (settlement <3000) | 0.81 | (0.59-1.11) | 0.183 |  |  |  | 14.4 |
|  |  |  |  |  |  |  |  |
| **ADMISSION CONTEXT** |  |  |  |  |  |  |  |
| Admissions in prior 1yr (per admission) | 1.05 | (0.96-1.16) | 0.253 |  |  |  | 10.6 |
| Length of stay (per week) | 0.99 | (0.95-1.03) | 0.593 |  |  |  | 0.4 |
| Emergency admission | 1.66 | (1.14-2.41) | 0.008 | 1.63 | (1.13-2.34) | 0.009 | 48.4 |
| Medical ward admission | 2.08 | (1.49-2.90) | <0.001 | 2.17 | (1.57-3.02) | <0.001 | 99.2 |
| Intensive care admission | 1.50 | (0.80-2.81) | <0.202 |  |  |  | 14.0 |
|  |  |  |  |  |  |  |  |
| **RENAL FUNCTION** |  |  |  |  |  |  |  |
| No AKI | (reference) | | |  | | |  |
| AKI stage 1 | 1.90 | (1.32-2.72) | 0.001 | 2.07 | (1.52-2.84) | <0.001 | 100 |
| AKI stage 2 | 3.41 | (2.11-5.49) | <0.001 | 3.68 | (2.40-5.63) | <0.001 | 100 |
| AKI stage 3 | 2.94 | (1.63-5.32) | <0.001 | 3.10 | (1.82-5.26 | <0.001 | 100 |
|  |  |  |  |  |  |  |  |
| Prior AKI count (per episode) | 1.18 | (0.96-1.44) | 0.121 |  |  |  | 23.8 |
|  |  |  |  |  |  |  |  |
| Baseline eGFR linear term^a^ | 1.00 | (0.75-1.35) | 0.993 |  |  |  |  |
| Baseline eGFR squared term^a^ | 0.98 | (0.96-1.01) | 0.193 | 0.98 | (0.98-0.99) | <0.001 | 99.0 |
|  |  |  |  |  |  |  |  |
| Discharge creatinine 20% > baseline | 1.03 | (0.71-1.49) | 0.891 |  |  |  | 2.4 |
|  |  |  |  |  |  |  |  |
| **COMORBIDITY** |  |  |  |  |  |  |  |
| Cancer | 0.71 | (0.43-1.17) | 0.176 |  |  |  | 4.2 |
| Cardiac failure | 4.68 | (3.43-6.38) | <0.001 | 5.45 | (4.15-7.17) | <0.001 | 100 |
| Cerebrovascular disease | 0.91 | (0.58-1.42) | 0.671 |  |  |  | 2.0 |
| Dementia | 0.43 | (0.15-1.21) | 0.108 |  |  |  | 2.2 |
| Diabetes | 1.08 | (0.74-1.58) | 0.678 |  |  |  | 3.2 |
| Hemiplegia | 0.36 | (0.05-2.76) | 0.329 |  |  |  | 0.0 |
| Liver disease | 0.92 | (0.28-3.04) | 0.894 |  |  |  | 0.6 |
| Myocardial infarction | 1.13 | (0.79-1.63) | 0.497 |  |  |  | 7.2 |
| Peptic ulcer disease | 1.14 | (0.59-2.22) | 0.703 |  |  |  | 3.2 |
| Peripheral vascular disease | 1.25 | (0.80-1.97) | 0.326 |  |  |  | 10.2 |
| Pulmonary | 1.04 | (0.70-1.53) | 0.859 |  |  |  | 2.4 |
| Rheumatic disease | 0.81 | (0.39-1.65) | 0.553 |  |  |  | 1.0 |
| Abbreviations: AKI, acute kidney injury; CI, confidence interval; eGFR, estimated glomerular filtration rate; OR, odds ratio. | | | | | | | |
| ^a^Modelled per 10ml/min/1.73m^2^ increase with linear and quadratic terms. The linear term can be included here but made no difference to the multivariable model (OR 1.00, p-value 0.998). Variable inclusion % applies to the baseline eGFR variable overall. | | | | | | | |
| ^b^In 500 bootstrapped datasets | | | | | | | |

Additional table S3 – Comparison of prediction models and model discrimination for death or readmission with pulmonary oedema after hospital discharge

|  | **Full model** | **Best stepwise model** | **Administrative data only model** | **Biochemistry  + age model** | **Age + sex alone model** | **AKI alone** |
| --- | --- | --- | --- | --- | --- | --- |
| **CHARACTERISTICS** |  |  |  |  |  |  |
| Age | * | * | * | * | * |  |
| Age term quadratic term | * |  |  |  |  |  |
| Male sex | * | * | * | * | * |  |
| Residential care | * | * | * |  |  |  |
| Deprived (highest quintile) | * |  |  |  |  |  |
| Rural (settlement <3000) | * |  |  |  |  |  |
|  |  |  |  |  |  |  |
| **ADMISSION CONTEXT** |  |  |  |  |  |  |
| Admissions in prior 1yr (per admission) | * |  |  |  |  |  |
| Length of stay (per week) | * |  |  |  |  |  |
| Emergency admission | * | * | * |  |  |  |
| Medical ward admission | * | * | * |  |  |  |
| Intensive care admission | * |  |  |  |  |  |
|  |  |  |  |  |  |  |
| **RENAL FUNCTION** |  |  |  |  |  |  |
| AKI stages 0-3 | * | * |  | * |  | * |
| Prior AKI count (per episode) | * |  |  | * |  |  |
| Baseline eGFR (linear and quadratic) | * | * |  | * |  |  |
| Discharge creatinine 20% > baseline | * |  |  |  |  |  |
|  |  |  |  |  |  |  |
| **COMORBIDITY** |  |  |  |  |  |  |
| Cancer | * |  |  |  |  |  |
| Cardiac failure | * | * | * |  |  |  |
| Cerebrovascular disease | * |  |  |  |  |  |
| Dementia | * |  |  |  |  |  |
| Diabetes | * |  |  |  |  |  |
| Hemiplegia | * |  |  |  |  |  |
| Liver disease | * |  |  |  |  |  |
| Myocardial infarction | * |  |  |  |  |  |
| Peptic ulcer disease | * |  |  |  |  |  |
| Peripheral vascular disease | * |  |  |  |  |  |
| Pulmonary | * |  |  |  |  |  |
| Rheumatic disease | * |  |  |  |  |  |
| **Model C statistic** | 0.857 | 0.853 | 0.837 | 0.811 | 0.735 | 0.642 |
| **95% confidence interval** | (0.836-0.878) | (0.831-0.874) | (0.815-0.859) | (0.787-0.835) | (0.710-0.760) | (0.611-0.672) |
| **P-value for AUC comparison with the next most complex model** | - | 0.143 | 0.005 | 0.034 | <0.001 | <0.001 |
| Abbreviations: AKI, acute kidney injury; eGFR, estimated glomerular filtration rate. | | | | | | |
