# Supplementary figures and images for: Acute kidney injury as an independent risk factor for unplanned 90-day hospital readmissions
Source: BMC Nephrol. 2017 Jan 6;18:9. doi: 10.1186/s12882-016-0430-4 (PMC5217258; doi:10.1186/s12882-016-0430-4)

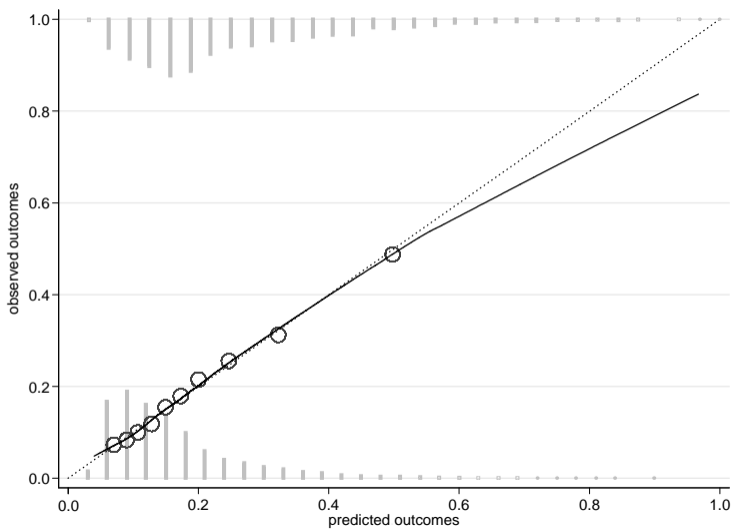

Supplement: Additional file 2: Figure S1. — Calibration plot for best stepwise prediction model of 90 day readmission or death. Circles represent observed and predicted readmission in deciles of predicted risk. Histograms represent the distributions of patients with (top) and without (bottom) readmission. (PDF 31 kb) [file 12882_2016_430_MOESM2_ESM.pdf]

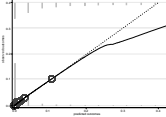

Supplement: Additional file 3: Figure S2. — Calibration plot for best stepwise prediction model of 90 day readmission with acute pulmonary oedema. Circles represent observed and predicted readmission in deciles of predicted risk. Histograms represent the distributions of patients with (top) and without (bottom) readmission. (PDF 40 kb) [file 12882_2016_430_MOESM3_ESM.pdf]

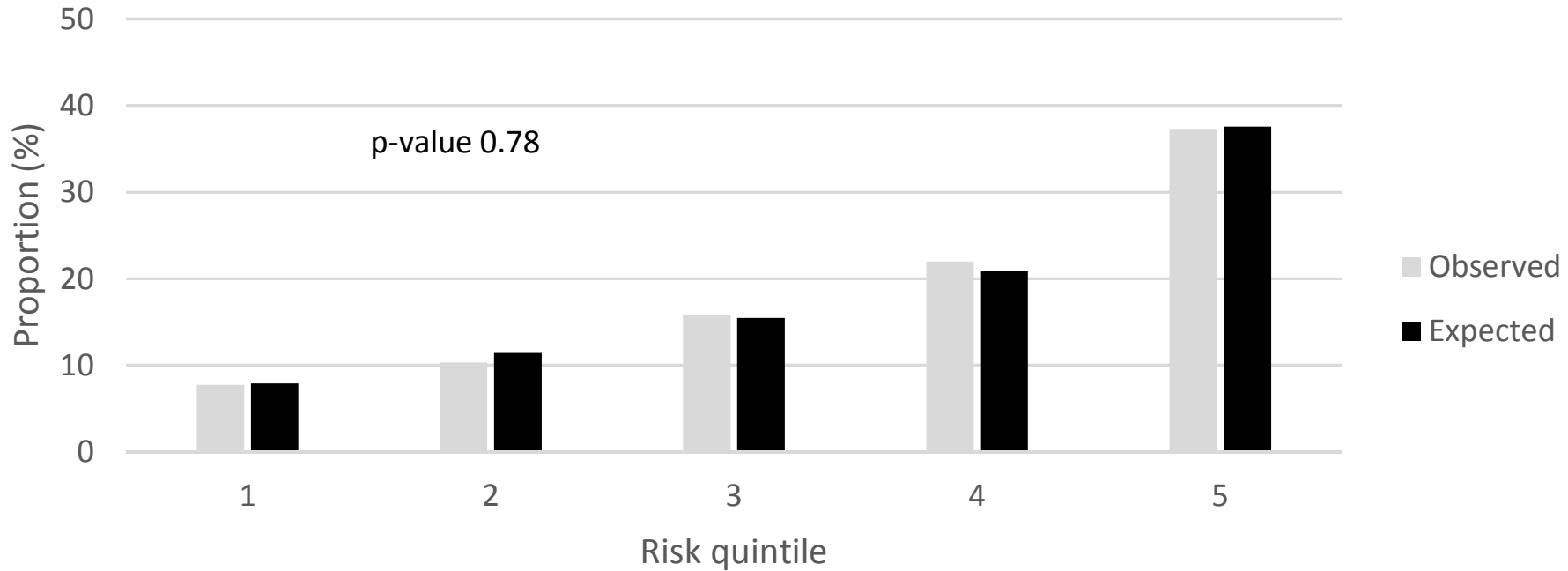

Supplement: Additional file 4: Figure S3. — Hosmer-Lemeshow test standardised for sample size for model of 90 day readmission or death with plots of quintiles of observed and predicted risk. (PDF 43 kb) [file 12882_2016_430_MOESM4_ESM.pdf]

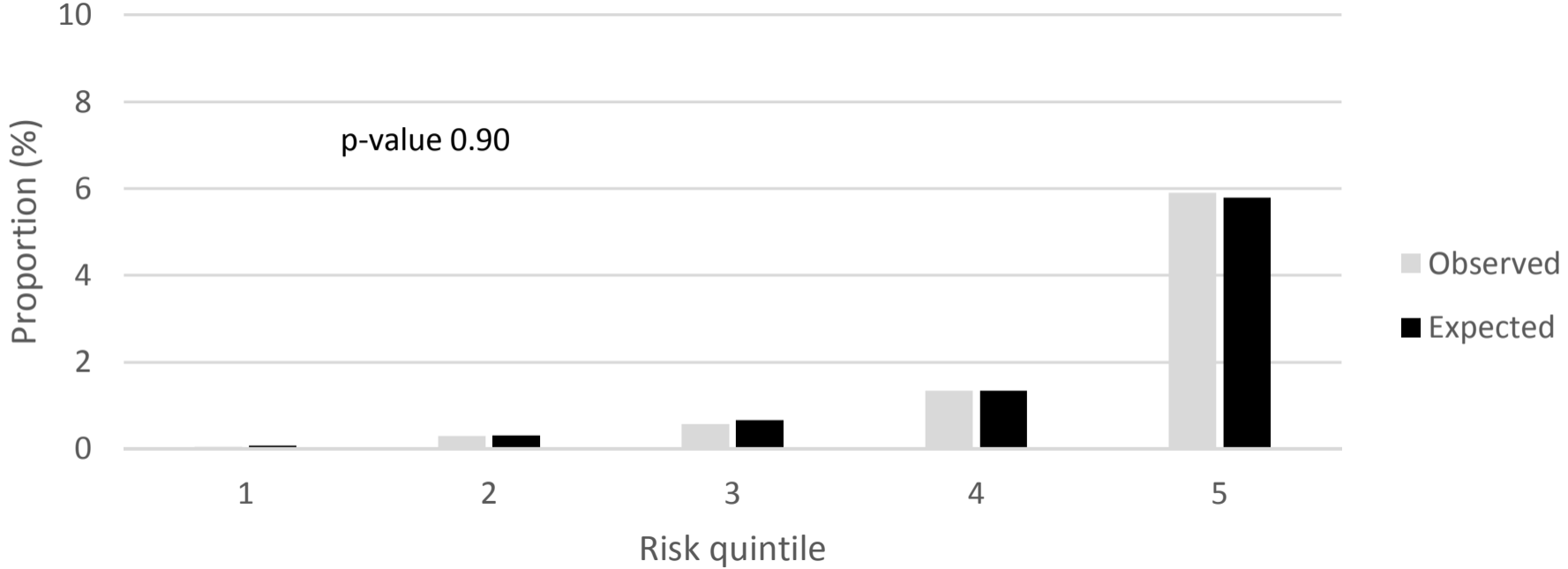

Supplement: Additional file 5: Figure S4. — Hosmer-Lemeshow test standardised for sample size for model of 90 day readmission with acute pulmonary oedema with plots of quintiles of observed and predicted risk. (PDF 43 kb) [file 12882_2016_430_MOESM5_ESM.pdf]
