# Supplementary material for: Acute kidney injury as an independent risk factor for unplanned 90-day hospital readmissions
Source: BMC Nephrol. 2017 Jan 6;18:9. doi: 10.1186/s12882-016-0430-4 (PMC5217258; doi:10.1186/s12882-016-0430-4)
Supplement: Additional file 6: Figure S5. — Illustration of clinical risk prediction model using examples with and without AKI. (PDF 647 kb) [file 12882_2016_430_MOESM6_ESM.pdf]

Aberdeen readmission risk calculator

Age

20 — 70 — 100

20 28 36 44 52 60 68 76 84 92 100

AKI severity stage (enter AKI stage 1-3 or no AKI)

AKI stage 3

Baseline eGFR

10 — 60 — 120

10 21 32 43 54 65 76 87 98 109 120

No. of previous admissions in past year

0 — 10

0 1 2 3 4 5 6 7 8 9 10

ADMISSION CONTEXT

(•) Emergency ( ) Elective

Residential care

(•) No ( ) Yes

Rural home location

(•) No ( ) Yes

PAST MEDICAL HISTORY

Cancer

(•) No ( ) Yes

Cardiac Failure

(•) No ( ) Yes

Diabetes

( ) No (•) Yes

Pulmonary disease

(•) No ( ) Yes

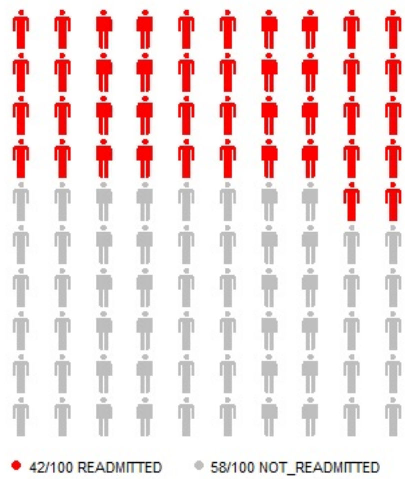

Out of 100 people discharged, this is the proportion expected to be readmitted or die within 90 days.

42

Aberdeen readmission risk calculator

Age

20 — 70 — 100

20 28 36 44 52 60 68 76 84 92 100

AKI severity stage (enter AKI stage 1-3 or no AKI)

No AKI

Baseline eGFR

10 — 60 — 120

10 21 32 43 54 65 76 87 98 109 120

No. of previous admissions in past year

0 — 10

0 1 2 3 4 5 6 7 8 9 10

ADMISSION CONTEXT

(•) Emergency ( ) Elective

Residential care

(•) No ( ) Yes

Rural home location

(•) No ( ) Yes

PAST MEDICAL HISTORY

Cancer

(•) No ( ) Yes

Cardiac Failure

(•) No ( ) Yes

Diabetes

( ) No (•) Yes

Pulmonary disease

(•) No ( ) Yes

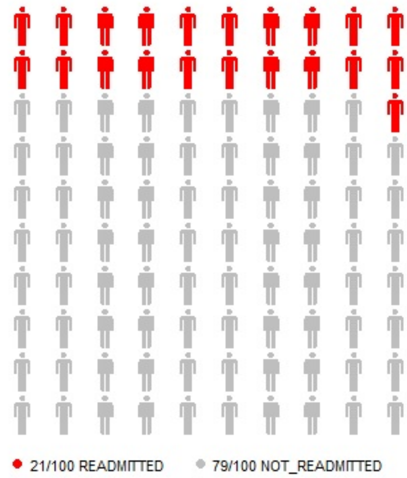

Out of 100 people discharged, this is the proportion expected to be readmitted or die within 90 days.

21
